# Biomolecules under Pressure: Phase Diagrams, Volume Changes, and High Pressure Spectroscopic Techniques

**DOI:** 10.3390/ijms23105761

**Published:** 2022-05-20

**Authors:** László Smeller

**Affiliations:** Department of Biophysics and Radiation Biology, Semmelweis University, Tűzoltó u. 37-47, 1094 Budapest, Hungary; smeller.laszlo@med.semmelweis-univ.hu

**Keywords:** high pressure, protein, lipid membrane, nucleic acid, G-quadruplex, FTIR, fluorescence, food science, deep sea

## Abstract

Pressure is an equally important thermodynamical parameter as temperature. However, its importance is often overlooked in the biophysical and biochemical investigations of biomolecules and biological systems. This review focuses on the application of high pressure (>100 MPa = 1 kbar) in biology. Studies of high pressure can give insight into the volumetric aspects of various biological systems; this information cannot be obtained otherwise. High-pressure treatment is a potentially useful alternative method to heat-treatment in food science. Elevated pressure (up to 120 MPa) is present in the deep sea, which is a considerable part of the biosphere. From a basic scientific point of view, the application of the gamut of modern spectroscopic techniques provides information about the conformational changes of biomolecules, fluctuations, and flexibility. This paper reviews first the thermodynamic aspects of pressure science, the important parameters affecting the volume of a molecule. The technical aspects of high pressure production are briefly mentioned, and the most common high-pressure-compatible spectroscopic techniques are also discussed. The last part of this paper deals with the main biomolecules, lipids, proteins, and nucleic acids: how they are affected by pressure and what information can be gained about them using pressure. I I also briefly mention a few supramolecular structures such as viruses and bacteria. Finally, a subjective view of the most promising directions of high pressure bioscience is outlined.

## 1. Introduction

Pressure is an often-overlooked physical parameter. In most cases, the thermal stability of biomolecules is examined, therefore, their conformational transition at elevated temperatures is well understood. Similarly, the effects of different chemical environments (such as pH, ionic strength, etc.) on the molecular conformation and function are also widely studied. Pressure, however, is rarely used in such investigations, although it is an equally important thermodynamic parameter as temperature. Technical difficulties could partially explain the rarity of pressure experiments. While proteins undergo a heat denaturation in a comfortably achievable range well below the boiling point of water, pressure effects are first visible above a few thousand times the atmospheric pressure. One can argue that this is highly non-physiological condition, but 80 °C and 8 M urea concentration is also quite far from the living conditions of ordinary biological systems.

Why is it worthwhile to perform high pressure experiments? There are several reasons: 1. since pressure is conjugated to volume in the thermodynamic relations, it is possible to obtain volumetric information about the system studied. 2. High pressure is not completely non-physiological, the biosphere spans up to 11 km in depth, which corresponds to more than 100 MPa (or one kilobar) pressure. A number of living organisms can be found in the deep sea, e.g., fishes were filmed even at 7–8 km depths [1]. The adaptation of these organisms is also an intensively studied question [2,3]. 3. Biophysical studies on biomolecules also led to several practical applications, mainly in the field of food science [4,5,6]. High pressure is used as a non-destructive technique in food processing in order to inactivate bacteria and increase the shelf life of the products [7].

The origins of the high pressure bioscience go back circa a hundred years. Bridgman was a high pressure physicist, who—out of pure curiosity—placed an egg white in his high pressure apparatus. As he noticed, treating the egg white for 30 min at 7 kbar “resulted in apparently complete coagulation” [8]. After this very first step, the field was silent for more than 50 years. High pressure bioscience gained interest again in the eighties and nineties of last century. The appearance of sophisticated spectroscopic and biotechnological equipment allowed researchers to follow the pressure-induced changes at the molecular level. It resulted in a revolutionary new approach and allowed researchers to obtain new information about a number of biological systems [9]. Several simple model systems were studied and characterized up to now, and volumetric aspects of simple and quite complex systems have been revealed [10,11,12,13,14]. Parallel to these discoveries of basic science, practical applications were invented too, mainly in the field of food science [5,15,16]. High-pressure food processing has become a reality, and high-pressure processed food is available now—not only for high pressure conference participants, but also in the shops as well. Another direction in high pressure food science research is the reduction of the allergenic character of food by pressure [17,18,19]. As mentioned earlier, high pressure naturally occurs in the biosystem, at the bottom of the deep sea. Understanding the adaptation to such an environment is also intensively examined [3,20,21].

With the appearance of new sophisticated microscopic techniques, new possibilities emerged for scientists interested in high pressure studies. Measuring single molecular changes under pressure is one of these cutting-edge applications of modern microscopic methods in the high pressure field [22].

This review summarizes the effect of pressure on biomolecules, starting from a thermodynamic description through the experimental approaches, reviewing the effects of pressure on the molecular level and on whole organisms, and finally I briefly deal with the practical applications of high pressure.

## 2. Pressure and Volume

Pressure and volume are conjugated physical parameters in thermodynamics. According to the Le Châtelier–Braun principle, the system changes its state in a way to reduce the external constraint. In the case of pressurization, this means that the system will adopt its smallest volume state. This principle can basically explain all pressure-induced effects; however, the actual conformational changes could be much more complex. Moreover, the definition of the volume of a molecule is not straightforward either. Indirect volumetric effects caused in the neighborhood of the molecule by electrostriction or by changing the hydration of the solute molecule should also be taken into account.

From an experimental point of view, the volume of a solute molecule can be defined using the principle of the partial molar volume [23]. The partial molar volume (*V_i_*) of a solute molecule is defined as the volume change (Δ*V*) of the solution by the addition of an infinitesimal amount of the solute (*i*) over the number of moles of added solute (Δ*n_i_*) keeping all the other thermodynamic parameters constant.

(1) $$V_{i}={\frac{\partial V}{\partial n_{i}}|}_{n_{j};p;T}$$

It is important to note that *V_i_* takes into account the interactions with the solvent, too. In order to explain the volume of the molecule at the microscopic level, all of the factors contributing to the change of the volume of the solution, when a solute molecule is added, must be considered. The major contribution comes from the volume occupied by the atoms of the molecule (*V*_a_). In the case of macromolecules, improper packing can lead to cavities inside the molecule (*V*_cav_). Only the cavities, which cannot be filled with water molecules, can contribute to the overall volume of the system. It is, however, not the only contribution, since the interactions with the solvent that can also influence the total volume must also be taken into account. The water layer around a macromolecule can be denser than the bulk water. This is taken into account by the hydration volume change (Δ*V*_hyd_), i.e., the change of the solvent volume due to the denser hydration layer. A further contribution comes from the thermal motion of the molecule, which is taken into account as the thermal volume (*V*_t_) [24,25].

*V_i_ = V*_a_ + *V*_cav_ + Δ*V*_hyd_ + *V*_t_ (2)

Unfortunately, it is very difficult to directly measure the above terms separately. Both the cavities and the hydration can play determining roles in the case of pressure-induced conformational changes, depending on the molecules in question, although the determining role of the cavities seems nowadays to be more accepted [14,26,27,28,29].

In order to achieve a measurable impact on the major biomolecules, one has to use a few thousand times the atmospheric pressure. This is because relatively small volume changes are associated with the conformational changes of these molecules. The upper limit of the experimentally available pressure range for biological molecules is determined by the freezing line of water, since biological samples mostly contain water as solvent. Pure water solidifies at 10 kbar (=1 GPa) at room temperature [30]. Highly concentrated solutions can stay liquid up to circa 15 kbar [31]. The pressure value that causes measurable changes in the studied system depends greatly on the volumetric properties of the system.

## 3. Pressure–Temperature Phase Diagram

As mentioned earlier, high pressure will favor those conformations that have smaller volumes. A two-state transition can be assumed in many cases. In order to describe these pressure-induced conformational transitions, one has to consider the difference of the Gibbs free energy values of the two states:

Δ*G* = *G*_2_ − *G*_1_, (3)

where *G*_1_ and *G*_2_ denote the Gibbs free energy in states 1 and 2, respectively. States 1 and 2 can be the folded and unfolded conformations of a protein, the gel and liquid crystal phases of a lipid layer, or differently folded (or unfolded) DNA conformations.

Either the integration of d(Δ*G*) = −Δ*S*d*T* + Δ*V*d*p* from a reference point of *T*_0_, *p*_0_ until a given *T*, *p* point [32] or a series expansion of Δ*G* [23] around *T*_0_, *p*_0_ will provide the following equation:

(4) $$\Delta G=\Delta G_{0}-\Delta S_{0}\left(T-T_{0}\right)-\frac{\Delta C_{p}}{2T_{0}}{\left(T-T_{0}\right)}^{2}+\Delta V_{0}\left(p-p_{0}\right)+\frac{\Delta \beta }{2}{\left(p-p_{0}\right)}^{2}++\Delta \alpha \left(p-p_{0}\right)\left(T-T_{0}\right)\;+higher\;order\;terms,$$

where the index refers to the value at *T*_0_, *p*_0_, *C_p_* is the heat capacity, and *α* and *β* are the thermal expansivity factor ($\partial V/\partial T{|}_{p})$ and the compressibility factor ($\partial V/\partial p{|}_{T})$, respectively. Δ always means the difference of the actual parameter between states 2 and 1 [30].

**Figure 1:**
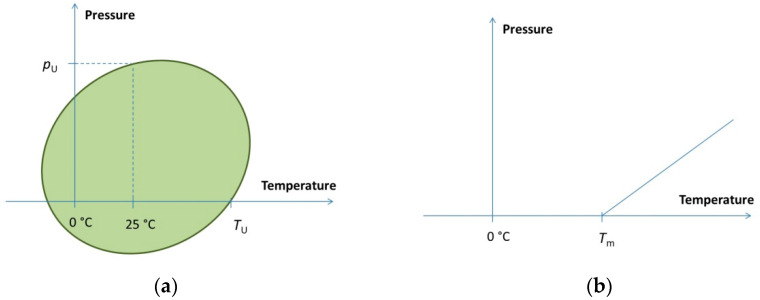
Pressure–temperature phase diagram types. (**a**) Elliptic phase diagram of proteins. Typical values are *p*_U_ = 200… 700 MPa, *T*_U_ = 50… 90 °C. (**b**) Linear phase boundary typical for lipid–water systems and for nucleic acid solutions. Typical slope for lipids is around 5 MPa/°C. *T*_m_ depends on the type and length of the chain and on the head group type. As an example, *T*_m_ = 41 °C for DPPC.

At the middle point of the phase transition Δ*G* = 0, the phase boundary in the pressure–temperature plane can be obtained from this condition. Depending on the actual thermodynamic parameters, the shape of the phase boundary curve can be elliptic (Figure 1a), such as in the case of proteins [23], or linear (Figure 1b), if the second order terms are negligible. The latter is typical for lipids and nucleic acids [33,34].

The mathematical condition for the elliptic shape is (Δ*α*)^2^ > ΔC_p_ Δ*β*/T_0_, which is fulfilled for the proteins. It is important to note, however, that a very big ellipse (or other second-order curve) cannot be experimentally distinguished from a simple line. The horizontal size of the ellipse (along the T axis) is defined by Δ*C*_p_. Small values of Δ*C*_p_ will result in an elongated ellipse. Similarly, the ellipse will be very much elongated in the pressure direction if Δ*β* is small. In these cases, one can practically observe a linear phase boundary such as the one in Figure 1b.

Although the higher order terms were neglected in Equation (4), it can be shown that the elliptic shape is not distorted considerably in the presence of moderate higher order terms [30].

A similar conclusion can be drawn from the Clausius–Clapeyron equation, which can now be written in the following form:

(5) $$\frac{\partial T}{\partial p}=\frac{\Delta V_{0}+\Delta \beta \left(p-p_{0}\right)+\Delta \alpha \left(T-T_{0}\right)}{\Delta S_{0}-\Delta \alpha \left(p-p_{0}\right)+\Delta C_{p}\frac{T-T_{0}}{T_{0}}}.$$

In the case of a linear boundary, one can obtain the volume change of the transition as follows:

(6) $$\Delta V=\frac{\partial T}{\partial P}\;\Delta S$$

This equation is useful if one performs temperature-scanning experiments at different pressures. In this case, Δ*S* can be obtained from the transition curve of the heating experiments. The measurable value (*y*), i.e., what is sensitive to the transition, can be fitted by the curve:

(7) $$y=aT+b+\frac{\Delta aT+\Delta b}{1+exp\left(\frac{\Delta H}{R}\left(\frac{1}{T}-\frac{1}{T_{m}}\right)\right)}.$$

Here *a*, *b*, Δ*a*, and Δ*b* are parameters of the linear change of *y* outside the transition region, *T_m_* is the mid-point of the transition, and Δ*S* can be obtained from the Δ*H*.

If one performs pressure-scanning experiments at constant temperature, the fitting of the transition curve allows the direct determination of the volume change (Δ*V*):

(8) $$y=ap+b+\frac{\Delta ap+\Delta b}{1+exp\left(\frac{\Delta V\;\left(p-p_{m}\right)}{RT}\right)}.$$

It must be noted that the use of these equations assumes a two-state transition, which is, in most cases, a reasonable approximation, even if short-living intermediates appear during the transition. The form of the transition is not restricted, it can be a protein unfolding, a lipid phase transition (e.g., the gel to liquid crystal one), the unwinding of a double helix DNA, or the unfolding of special nucleic acid structures such as G-quadruplexes. In Equations (7) and (8), *y* can be any measurable physical quantity, which reflects the amount of the low temperature phase or structure.

## 4. High Pressure Techniques and Spectroscopic Methods Used in High Pressure Bioscience

### 4.1. Instrumentation

**Figure 2:**
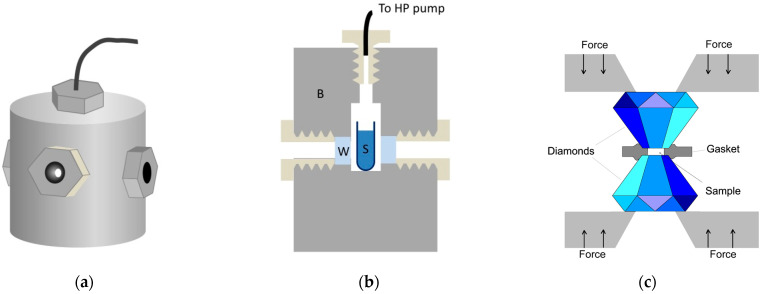
High pressure instrumentation. (**a**,**b**) View and structure of the thick-wall cylinder-type high pressure optical cell. B: cell body, W: optical window S: sample solution. (**c**) Schematic view of the diamond anvil cell.

As it was mentioned before, the relevant pressure range for typical biological molecules is up to 1 GPa, in some special cases up to 2 GPa. Basically, two completely different methods are in use to produce such pressures: 1. thick-wall cylinder, sometimes called a “pressure bomb”, 2. the diamond anvil cell (DAC) [35]. They are schematically presented in Figure 2.

The thick-wall cylinder system contains a high-pressure pump that is coupled by special tubing to an optical cell. This cell—as its name suggests—has a few-centimeters-thick wall in order to withstand the inner pressure of typically 0.4–0.6 GPa. The wall can be stainless steel or a copper-beryllium alloy, the latter provides a good combination of strength and thermal conductivity [36]. Typical sample volumes of this type of cell designed for optical experiments are few cm^3^. The most critical parts of this system are the optical windows. Their fitting and the tightening of the sealing is crucial for the appropriate operation of the system. For most applications, sapphire is used as the window material, because it is hard and transparent from 230 nm to 4 μm. Pressure is measured usually by a Bourdon gauge attached to the tubing between the pump and the optical cell. Electronic transducers operating with a wire strain gauge are also available.

A completely different principle is used in the case of DAC [37]. Two diamond anvils are used as optical windows. The tops of the anvils are cut, in order to obtain two parallel surfaces for the windows. Apart from the two diamonds facing each other, there is only one additional component in the DAC: the gasket. The gasket is a thin (50–200 μm thick) plate, with a hole in the middle. This hole contains the sample, and the remaining plate supports the pressure inside (see Figure 2b). Typically, the hole has a diameter of 0.5 mm, the sample volume can be as small as a few tens of nanoliter. The gasket is indented before filling the DAC for better sealing. Pressure is produced by pushing the two diamonds together using a lever arm system or a pneumatic system or by simply screwing them together. Special forms of DAC are capable of producing pressures of up to a few 100 GPa, which is used by solid state physicists. The pressure must be measured by an internal calibrant. Ruby fluorescence, infrared bands of quartz, and BaSO_4_ are regularly used [38,39,40,41,42]. These optical signals show slight spectral shifts along with the pressure, which allows the calculation of the internal pressure of the DAC.

### 4.2. Absorption Spectroscopic Techniques

#### 4.2.1. UV-VIS Absorption Spectroscopy

Focusing on the three main biomolecule types: protein, lipid, and nucleic acid, the question is, which part of the molecule is absorbing and what kind of information can be obtained from the changes of the absorption band? The absorption of proteins originates from the three aromatic amino acids [43] and from the prosthetic groups if there are any [44]. The amino acid residues absorb in the UV range, with absorption maxima of 280, 274, and 257 nm for tryptophan, tyrosine, and phenylalanine, respectively. Since the spectral bands are quite wide and overlapping, fourth-derivative spectroscopic evaluation was developed [45]. This technique is mainly used in high pressure food-related research [46].

UV absorption spectroscopy is widely used for nucleic acids. The unwinding of the double helical DNA can be easily seen using the hypochromic effect at 260 nm [47,48].

It must be mentioned, though, that circular dichroism spectroscopy, which is the most standard method of biophysics for secondary structure determination, cannot be used in high pressure research. The reason is the birefringence of the windows, which changes along with the pressure. This obscures the weak CD signal, and the attempts of its compensation or correction have failed. Therefore, alternative methods for secondary structure determination, such as infrared spectroscopy and Raman spectroscopy, gained importance.

#### 4.2.2. Infrared Absorption Spectroscopy

Infrared spectroscopy provides information about molecular vibrations. Since molecular vibrations are sensitive to such changes in the environment of vibrating atoms, such as conformational changes or formation of a hydrogen bond, spectral shifts of specific vibrational bands can be used to follow the conformational changes of the molecule. It is also suitable for lipid systems [49], proteins [50], and nucleic acids [51]. Lipids show strong C-H vibrational bands in the range of 2800–3000 cm^−^^1^. Among them, the two most intensive are the symmetric and asymmetric stretching vibrations of the CH_2_ groups at around 2850 and 2920 cm^−^^1^. Since the wavenumber calibration of the modern FTIR spectrometers is very precise, the phase transition can be measured even if the frequency shift of the vibrations corresponds with only 2 cm^−^^1^. Moreover, the CH modes, the C=O stretching at 1735 cm^−^^1^, and the stretching vibrations at 1243, 1090, and 1065 cm^−^^1^ associated to the phosphate group are the most frequent markers of the changes in the lipid phase.

The amide I vibration is the most conformation-sensitive band of the infrared spectrum of proteins. It is mainly a C=O stretching of the protein backbone. Due to the delocalized peptide bond, the position of the band is at a lower frequency than the C=O stretching of the lipids. The amide I band of the proteins can be found in the range of 1600–1700 cm^−^^1^. The frequency of the amide I vibration is slightly different for each secondary structure type (α-helix, β-sheet, unordered structure, etc.) [52,53]. In a real protein, however, several secondary-structure types are present, which results in a broad amide I envelope spectrum, composed of overlapping component bands of the secondary structure elements. Mathematical methods, such as Fourier self-deconvolution and band fitting, are necessary to obtain quantitative (or at least semi-quantitative) information about the secondary-structure contents of proteins [52]. Since the internal hydrogen bonds are missing in the unfolded protein, and they are substituted by protein–water hydrogen bonds, upon unfolding of the protein, the amide band becomes broad and featureless. Special “side bands” at 1616 and 1685 cm^−1^ are characteristic of the intermolecular β-type interactions, i.e., for the aggregation of proteins [54].

Since the amide I band i overlaps with the strong bending vibration of the water, heavy water (D_2_O) is routinely used for these experiments. While the amide I band is practically insensitive to the hydrogen-deuterium (H/D) exchange, the amide II band contains a large contribution from the N-H bending motion, which results in a circa 100 cm^−^^1^ wavenumber shift upon deuteration of the protein. This allows scientists to follow the H/D exchange in proteins. This also reports the solvent accessibility of the given part of the polypeptide chain, because the buried part of the folded protein remains unexchanged for a long time in the D_2_O-based solution. Loosening of the tertiary structure allows penetration of the solvent and enhances the exchange of the hydrogen atoms in the interior of the protein structure. This means that, while the amide I band allows following the secondary structural changes, the amide II band reports the tertiary structural changes [55,56].

The pressure shift of the spectral lines in vibrational spectroscopic techniques depends on the contribution of the normal vibration to the volume of the molecule [57]. Typically, dν/dp values are positive, due to the bond strengthening upon pressurization. They are large for breathing vibrations, while antisymmetric stretching modes, which hardly influence the volume, are pressure insensitive. The vibration involving hydrogen bonding is a special case: an example is the amide I vibration of a folded protein. In this case negative dν/dp values can be measured. The reason is that pressurization shortens the H-bond; therefore, the electron density increases around the proton, resulting in the decrease in the electron density of the CO bond. Thus, the CO bond will be weaker, and the vibrational frequency will decrease.

One must be careful, however, if the band is composed of several sub-bands, such as in the case of the amide I band of the proteins. The amide I band is composed of sub-bands representing the secondary structure types [58]. In this case the position of the overall band is not the correct parameter to characterize the transition. One must follow the absorbance changes in the positions of the individual sub-bands. An alternative is to fit the sub-bands, which is mostly completed after increasing the resolution by a self-deconvolution procedure [59].

### 4.3. Fluorescence Spectroscopy

The intrinsic fluorescence of proteins originates from the three aromatic residues. Tryptophan is used in most studies since it has the highest fluorescence emission intensity. Sometimes, in the absence of tryptophan, the weak tyrosine signal is also measurable.

Tryptophan fluorescence emission is very sensitive to the polarity of the environment, which makes it optimal for the detection of a protein with buried tryptophan residue(s) [60].

The green fluorescence protein is widely used nowadays in cell and molecular biology. Its genetically engineered variants provide a series of fluorescence proteins, covering the whole visible spectrum [61,62]. Their beta barrel structure turned out to be very pressure-resistant, and the chromophore in the middle of the barrel is quite well preserved from the influences of the environment, which makes them a suitable marker for pressure studies of various cellular systems [63].

The intrinsic fluorescence of nucleic acids is extremely weak; their quantum yield at room temperature is very low, in the order of 10^−4^ [64]. Therefore, instead of their intrinsic fluorescence, the most widely used methods for characterizing nucleic acids are absorption spectroscopy and fluorescence labeling.

FRET (Förster Resonance Energy Transfer) is a special fluorescence method used to detect conformational changes by measuring distances at the molecular level (3–10 nm). Two fluorophores are needed for this method: one is called the donor, and the other is the acceptor. When the donor is excited, an energy transfer phenomenon can be observed to the acceptor if the chromophores are within a so-called Förster distance (characteristic of the chromophore pair). As a result, the acceptor emits light, while the donor’s emission reduces. If the chromophores are far apart from each other, only the donor emits. Using this method, one can determine the folding of a macromolecule or the association of two molecules. This method was successfully applied for the detection of the folding of special nucleic acid structures, G-quadruplexes [65].

Nowadays, the evolution of microscopic techniques makes it possible to observe FRET at single molecular level (smFRET). An extremely diluted sample is used for this method, and the labeled molecules are usually fixed on a surface. The smFRET method allows us to observe the differently folded states of the studied molecules. These states were not possible to observe earlier when the spectra of the whole solution were recorded and analyzed. smFRET also works under pressure, using high pressure optical cells for microscopy [66]. An alternative is a thin capillary, which is able to withstand pressures up to 500 MPa [67,68].

### 4.4. Raman Spectroscopy

Raman spectroscopy is sensitive to molecular vibrations, similar to the infrared spectroscopy. It can be easily used for detecting lipid phase transitions, due to the intensive bands of the CH vibrations, but it can serve for determining the protein conformational changes too.

Although the weakness of the Raman scattering limits its applications in the structural analysis of biomolecules, there are some recent examples where it was successfully applied in the high pressure field. It was used to study pressure’s effect on collagen [57], and another group used Raman spectroscopy to detect the germination of pressure-treated spores by following the 1017 cm^−^^1^ band of the Ca-dipicolinic acid [69].

Yang et al., studied the effect of pressure on the four nucleotide bases using Raman and infrared spectroscopy techniques. They observed spectral shifts in the range of −0.07–0.91 cm^–1^/kbar for guanine [70].

### 4.5. Nuclear Magnetic Resonance (NMR) Spectroscopy

Nuclear magnetic resonance spectroscopy is a very powerful tool for studying proteins. One of the pioneers of high-pressure NMR was Jonas, who studied the unfolding of apo-myoglobin and the pressure-assisted cold denaturation of ribonuclease [71,72]. At the same time, Akasaka’s group on the other side of the world also conducted high-pressure NMR studies on proteins [73,74]. Several groups apply this technique nowadays [75,76,77,78,79,80].

Technically, the most challenging part is the construction of a high-pressure cell that will not degrade the high resolution of the spectrometer. The best results are achieved nowadays using ceramic high-pressure cells [81].

The main advantage of the modern 2D NMR methods is that the cross-peaks of the spectrum can be assigned to certain amino acids. This way, the conformational changes can be followed on the level of the amino acids. Distortions of the macromolecule caused by sub-denaturing pressure values can be observed. These can be informative about the conformational fluctuations of the molecule in its native state [82]. Furthermore, the transition in the tertiary structure can be followed by measuring the H/D exchange of the individual residues.

NMR can be used to determine the 3D structure of non-helical nucleic acid forms. It also must be mentioned that the problem of polymorphism of G-quadruplexes can be resolved quite effectively using NMR [83,84,85]. These measurements were performed at atmospheric pressure, but, since G-quadruplexes are pressure-sensitive, there might be high potential in this field.

## 5. Pressure Effect on Some Biomolecules

### 5.1. Proteins

Pressure behavior of several water-soluble proteins has already been investigated [86]. Generally speaking, proteins usually unfold at room temperature under 400–600 MPa, although very weak ones can unfold at lower pressures, and there are such proteins which can preserve their native structure even above 1 GPa. These are, e.g., heat shock proteins and beta barrel proteins, [31,63]. The typical shape of the phase diagram (as mentioned before) is an ellipse, with the native state in the middle (Figure 1a). The three typical ways of crossing the ellipse are heat, pressure, and cold denaturation. The thermodynamic description of the elliptic diagram was first given by Hawley [32].

The complete pressure–temperature diagram was determined in the cases of several proteins; one of the first ones was myoglobin. Kauzmann determined its p-T phase diagram at different pH values [87]. Although the diagram was not completely elliptic, due to technical difficulties and slow relaxation times at high pressures and temperatures, it resembled the elliptic form. The native phase was clearly shrinking when the pH deviated from neutral. This latter effect seems to be a general feature for all proteins, and it helps the scientists, who are unable to reach the high enough pressures to denature the proteins; they can simply lower the pH to bring the unfolding pressure into the experimentally available pressure range [88]. An alternative method is to use the sub-denaturing concentration of urea or guanidine hydrochloride. After the initial work by Kauzmann, which was performed by absorption spectroscopy, several other methods were used to look into the details of the phase diagram [19,89,90]. Elliptic or elliptic-like phase diagrams were found for several proteins: myoglobin, lysozyme, red-shifted GFP variant, parvalbumin, staphylococcal nuclease, etc. [19,55,61,90,91].

Another very important factor influencing the phase diagram is the presence of intermolecular interactions. Several techniques, such as tryptophan fluorescence and UV-VIS absorption spectroscopy use low concentration samples (μM or less). On the contrary, FTIR and NMR spectroscopic methods need a few orders of magnitude higher concentrations. In the case of FTIR, 20–70 mg/mL concentrations are routinely used, which correspond to a few mM concentration of a monomeric protein. At these concentrations, the intermolecular interaction cannot be ruled out. My experiments show that intermolecular interactions play a role almost exclusively, if a water-soluble protein is unfolded or the native structure is at least partially lost (misfolded protein). Aggregation of such misfolded proteins appears at the phase diagram [92]. Meersman et al. provided the complete phase diagram of myoglobin, including the partially folded and aggregated states [89]. It has also been shown that the intermediate states, which can be populated after a pressure unfolding cycle, have higher aggregation propensities compared with the correctly folded native state. These aggregates were also proven to be pressure sensitive. A pressure of 200 MPa prevented the formation of the aggregates, and pressure had effectively dissociated the aggregated myoglobin structures [92]. Similar behavior was found in the case of lysozyme and human serum albumin [55,93]. Misfolded protein states and their aggregation play a crucial role in a series of so-called conformational diseases [94,95,96,97,98,99]. It turned out that the formation of the fibrillar aggregates, which plays role in the amyloidogenic diseases, needs a maturation time. Protein aggregates are formed first in amorphous forms, which convert to fibrillar ones in a few days [100]. Unfortunately, the matured fibrillar structures are quite resistant to pressure and temperature; they cannot be dissociated by pressures up to 1.3 GPa [100].

As was mentioned before, the pressure stability of proteins is very diverse, depending on the primary and secondary structures, as well as on the stabilizing factors of the tertiary structure. Disulfide bonds, bound ions, or ligands can play an important role in increasing stability. An example of a pressure sensitive protein is an apple allergen, called Mal d1; 200 MPa was able to unfold this protein. On the contrary, horseradish peroxidase (HRP) is a very pressure-resistant protein (p_unfolding_ = 1.2 GPa). The effect of Ca^+^ removal, i.e., the reduction of the disulfide bonds and heme substitution on the pressure stability of HRP, was studied by high-pressure FTIR spectroscopy. All these modifications only slightly reduced the stability; even in the most destabilized form, the reduced one is stable up to 0.95 GPa [101].

Another example of an extremely pressure resistant protein is the heat shock protein (HSP16.5) derived from the thermophilic archaebacteria Methanococcus jannaschii, which denatures at 1.7 GPa [31].

In the case of enzymes, not only is the stability important, but their flexibility is also a key factor. It is well known that enzymatic activity needs a certain degree of structural movement, which is possible due to the dynamic fluctuations of the protein. The fluctuations and compressibility are connected by the fluctuation–dissipation theorem of statistical physics. The compressibility of proteins can be obtained from various methods: volumetric experiments, ultrasound velocity measurements, site selective hole burning, or fluorescence line narrowing spectroscopies [102,103,104,105,106]. There is a delicate balance in the proteins between the flexibility needed for the enzymatic activity and the rigidity needed for preserving the stable structure [20]. Interestingly, this balance is shifted in the case of thermotropic or barotropic enzymes to preserve the optimal flexibility in the preferred environment.

The role of proteins in the pressure adaptation of deep-sea organisms is an active research field with both basic scientific and technological importance [107]. Some of the extremophile enzymes are already in use in industrial processes [108]. The use of modern genomic tools opens new perspectives in this field. Comparison of the expression pattern of piezophilic and mesophilic organisms provides new insight into the pressure-adaptation mechanisms. The pressure-shock-induced alteration of the complex metabolic and genetic networks in eukaryotic cells was studied in detail in the case of S. cerevisiae at the molecular level by Abe [3,109]. Morris et al. found significant increases in the transcription of genes coding GAPDH and the heat shock protein HSP70 in response to high pressure [110]. Sieg et al. compiled a database comparing protein sequences from deep-sea organisms with their orthologous proteins [111]. As mentioned previously, the stability and flexibility of the deep-sea proteins should change on the way to allow them to be able to function under pressure. A clear pattern or mechanism for high pressure adaptations was, however, not apparent from the database [111]. Actin is one of the most basic proteins in all living organisms. Globular actin is stable up to 270 MPa at room temperature [112], but polymerization of the actin monomers is known to be a very pressure-sensitive process; even pressures of a few hundred bar lead to a significant deceleration of the polymerization reaction, mainly due to the prolonged nucleation phase [113]. Morita found amino acid substitutions which can also contribute to the deep-sea adaptation of actin [114].

Moreover, sub-denaturing pressures can affect the conformation of enzymes. Enzymatic function is influenced this way, especially if the active site is distorted. The inactivation and activation of different enzymes in fruit juices, pulp, and purées using high-pressure treatment was studied by Roobab [115]. Hendrickx‘s lab measured the activity of several food-relevant enzymes as functions of pressure [116]. In the case of carrot pectinmethylesterase, where the whole T-p plane has been explored, the enzymatic activity showed similar elliptic-like character to the stability diagram of the proteins. Interestingly, the maximal activity was achieved under an elevated pressure of 350 MPa [117].

While there is much experimental evidence for the pressure denaturation of water-soluble proteins, much less is known about the pressure behavior of membrane proteins. A recent paper reports the denaturation of the crucial part of the light-harvesting 2 integral membrane complexes of purple photosynthetic membrane under a pressure of 400 MPa [118].

### 5.2. Lipids

**Figure 3:**
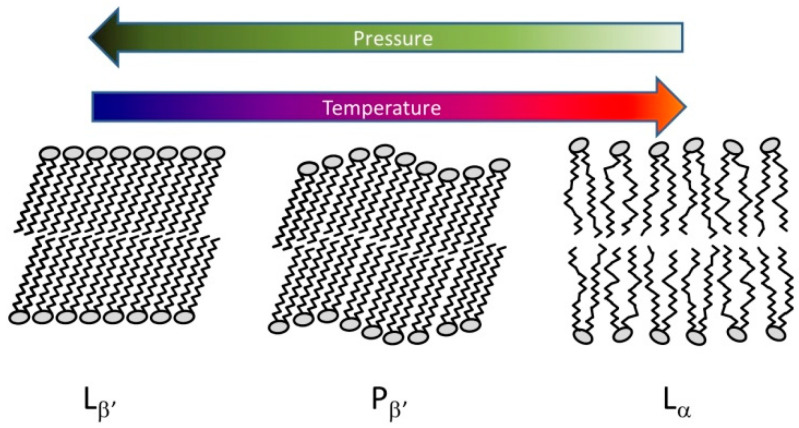
Typical lamellar lipid structures. L_β’_: gel phase with tilted chains, P_β’_ periodic rippled structure with tilted chains, L_α_: fluid-like liquid crystal phase.

Due to their amphiphilic character, lipids form various structures in aqueous media [119]. The simplest form is the lamellar bilayer. Different ordering of the hydrocarbon chains results in the temperature-dependent formation of lamellar gel or the fluid-like liquid crystalline phase (Figure 3). In some cases, a third rippled phase arises just below the main transition into the liquid crystalline phase.

A number of model membrane systems (containing only one type of lipid molecule) were measured and characterized by Wong at the end of last century using Raman and infrared spectroscopy [11]. He found the stabilization of the gel phase at elevated pressure, with a typical dT_m_/dp = 0.2 °C/MPa for phosphatidylcholine-type lipids. The phase transition lines of lipids with different chain lengths were parallel and shifted to each other according to their different phase transition temperatures at atmospheric pressure. The phase transition from the planar fluid phase to the inverse hexagonal (HII) phase of lipid–water systems is much more sensitive to pressure because of the high ΔV [119].

Densitometry and small angle x-ray scattering (SAXS) were applied by Winter’s group to characterize the volumetric properties of lipid membranes. A total of 3% volume increase has been measured in the case of DMPC (dimyristoylphosphatidylcholine) at the phase transition at 24 °C. This corresponds to 27 cm^3^/mol, which is considerable compared to proteins. Despite the relatively small (<1 kDa) molar mass of the lipid, compared to the few tens of kDa of the proteins, the latter ones have only a few times larger unfolding volume change. The relatively high-volume increase during the gel-to-liquid crystal phase transition is due to the increased void volume in the region of the hydrocarbon chains. It accounts for the high dT_m_/dp value of the lipids.

Lipid phase diagrams are strictly linear, at least in the region of the first few 100 MPa. They do not show any sign of having an elliptic character. A curved phase boundary has only the interdigitated gel phase, which appears at high pressures and temperatures (>50° and 150 MPa for DPPC) [119]. A similar interdigitated phase was found by high pressure NMR experiments for DPPG (dipalmitoylphosphatidylglycerol) bilayers [120].

The role of chain unsaturation was investigated by Skanes et al. [121]. They obtained a smaller (0.13 °C/MPa) dT_m_/dp value for PLPC (palmitoyl-2-linoleoyl-sn-3-glycero-phosphocholine, 16:0–18:2 PC) at the usual 0.20 °C/MPA that was observed for the saturated chain phosphatidylcholines earlier. This suggests that increasing the level of chain unsaturation reduces the sensitivity of the bilayer order to pressure.

As the simple systems have been characterized, the interest of researchers interested in high pressure studies turned to specific lipid types [122], lipid–protein interactions, and specific environmental effects, such as low hydration and the effect of cosolvents, etc. [123].

The interaction of Gramicidin D (an antibiotic polypeptide) with membranes was also widely studied [124,125]. The influence of Gramicidin D incorporation on the structure and phase behavior of DMPC lipid bilayers was studied using SAXS and NMR spectroscopy. The insertion of gramicidin into the DMPC membrane resulted in the broadening of the main-phase transition, even at a less than 5% molar ratio of gramicidin. Simultaneously, the slope of the phase-transition line on the p-T phase diagram was reduced to 0.16 °C/MPa. In the presence of gramicidin, a narrow fluid–gel coexistence region was formed instead of the sharp transition [124].

The importance of hydration was presented in the experiments by Heremans’ group [126]. Low hydrational levels decreased the pressure of the liquid crystal to gel phase transition, stabilizing the gel phase of the lipids. It is interesting to note that a similar effect was found for proteins by the same group; the folded state was stabilized at low hydration levels. These findings underline the importance of the hydration layer and the hydrogen bonds between water and the solute molecule in the pressure stabilization.

The curvature of the lipid membrane also plays a role, since it influences the volume and the tightness of the packing [127].

Special lipids appear in organisms living in extreme conditions. Bipolar tetraether liposomes created from the polar lipid fraction of thermoacidophile archaeon Sulfolobus acidocaldarius have been studied by FTIR spectroscopy and SAXS [122]. Tetraether lipids were also shown to form stable black lipid membranes [128]. Although the whole phase diagram was not determined, a pressure-induced phase transition was detected from the shift of the symmetric CH_2_ stretching mode. This appeared at 8.0 kbar at 60 °C, and 10.8 kbar at 20 °C, showing an unusual negative dT_m_/dp value. The authors explain this phenomenon with the presence of the hydrogen bond network in the polar headgroup region that weakens as the temperature increases.

TMAO (trimethalamine-N-oxide) is known to stabilize proteins against various stress factors, including high pressure. Winter’s group showed that TMAO also has marked effect on lipid bilayer systems [21]. TMAO shifted the gel-to-fluid phase transition to a higher temperature, stabilizing the gel phase, as seen from the high-pressure experiments.

One of the most important directions of the lipid-related field might be the investigation of the extracellular vesicles. They are used in cell-to-cell communication, and they are promising as next-generation drug delivery platforms [129]. Although there is no publication in this field that studies the effects of high pressure, this could be an interesting direction for the renaissance of the high-pressure lipid research.

### 5.3. Nucleic Acids

Early measurements found that nucleic acids are relatively pressure insensitive. Macgregor et al. measured the volume change of the helix–coil transition of poly[d(AT)], poly(DA)poly(dT), and poly[d(CG)] sequences [130]. Their ΔV values were very sensitive to the NaCl concentration; higher concentrations increased the ΔV values, but the ΔV values were smaller than 10 cm^3^/mol even in the presence of 1 M NaCl. Another investigation reported that Δ*T*_m_/Δ*p* values (where *T*_m_ is the temperature of the helix–coil transition) are dependent of the ionic strength. A 29 °C/GPa pressure shift was measured for 0.2 M ionic strength in the pressure range of 0.1–400 MPa [131]. The helix conformation was preferred by the pressure at all ionic strength conditions, indicating that the helix conformation has smaller volume. The calculated unfolding volume changes were less than 4 cm^3^/mol. This is the reason why nucleic acids fell out of the focus of researchers for a long time, i.e., due to their moderate pressure sensitivity.

However, noncanonical forms of nucleic acids have recently come to the attention of researchers using high pressure techniques. Among them, the four-strand G-quadruplex (GQ) structures are the most important ones [132,133]. They are formed by the guanine-rich sequences of the genome; four guanines are arranged in a planar structure stabilized by Hoogsteen-type hydrogen bonds. Two or three guanine quartets compose a G-quadruplex, and, in the middle of the GQ, monovalent cations are coordinated. The most effective stabilizers are K^+^ and Na^+^ ions, but GQs stabilized by Rb^+^ or Li^+^ have also been reported [65,134,135,136,137]. GQ structures are polymorphic depending on the sequence, metal ions, and the co-solute [138,139]. As for the orientation of the strands, they can appear in parallel, antiparallel, or mixed forms [136]. The four nucleic acid strands can belong to the same molecule (intramolecular GQ) or to two or four molecules (intermolecular GQs). GQs can also interact with several proteins [140]. DNA GQs aroused great interest in the field of cancer research when they were found in the telomere region and in several oncogene promoter regions. The formation of GQ structures in the telomere region inhibits the telomerase enzyme, which elongates the telomere region in the immortal (cancer) cells [141]. These discoveries made GQs promising targets for cancer research [142].

GQs turned out to be much more pressure sensitive than the double helical forms. A series of papers has recently been published in this field, which shows the renaissance of the high-pressure nucleic acid field [25,143].

The first pressure investigation of GQs was performed on the telomere repeat (Htel) [25]. The unfolding temperature *T*_m_ increased with increasing Na^+^ ion concentrations in the range of 20–100 mM. *T*_m_ decreased along with pressure, indicating the higher volume of the folded GQ form. The d*T*_m_/dp value was about −0.1 °C/MPa, and Δ*V* = 56 ± 2 cm^3^/mol was found at 100 mM Na^+^ ion concentration. According to the calculations of the authors, the release of water molecules from the hydration shell upon folding is responsible for the higher volume of the folded form.

Sugimoto’s lab investigated the thrombin binding aptamer (TBA), which is one of the shortest sequence-forming GQs [143,144]. It is composed of 15 bases and forms a two-quartet GQ. Although the two-quartet GQ is expected to be weaker in stability, but it was compensated for by the stronger binding of the potassium ion used in this study. The unfolding volume change was found at −55 ± 5 cm^3^/mol, which decreased dramatically in the presence of crowding agents such as ethylene glycol and PEG (polyethylene glycol). The volumetric contributions of loop regions of TBA were also analyzed in detail by the same group [145].

The high pressure and volumetric behavior of several other GQs, among them the c-MYC, KIT, and VEGF, were characterized. These GQ-forming sequences can be found in the promoter regions of cancer-related proteins. High-pressure infrared and FRET experiments revealed small volume changes of 6–18 cm^3^/mol [34]. Pressure decreased the unfolding temperature in all three cases, indicating the higher volume of the folded state.

The relatively high sensitivity of GQs to pressure emphasizes the importance of the deep-sea adaptation of these forms. Winter’s lab investigated the effect of TMAO, which is known as a stabilizer in deep-sea organisms. TMAO was found to protect the antiparallel conformation of the human telomeric GQ from pressure stress [68].

Sugimoto’s group characterized the volumetric aspects of the binding of hemin to Htel. They obtained significantly different binding volumes in the presence of Na^+^ and K^+^ ions (2.5 and −41.7 cm^3^/mol, respectively) [146]. Interestingly, the presence of the crowding agent PEG200 reduced the destabilizing effect of the pressure on the GQ-hemin complex.

Naturally, not only the human genome contains GQ-forming sequences; several viral genomes were analyzed, and the potential targets of the antiviral therapy were reviewed by Ruggiero [147,148]. The genome of the SARS-CoV-2 virus has also been analyzed from this point of view [149], and some of the predicted viral GQs were experimentally found [148,150]. In my previous papers I analyzed three GQ-forming sequences of the Hepatitis B virus [65,151], and genomic analysis suggested the possible GQ formation of these sequences [152,153]. Using FTIR and FRET measurements, I proved that all these three sequences (called HepB1, HepB2 and HepB3) form GQ structures. I also stabilized these GQs with ligands developed for targeting human GQs to achieve therapeutic effect for cancer. Volumetric properties were also revealed using my special high-pressure cell for fluorescence experiments. ΔV = 17 cm^3^/mol was found for HepB1, while the two others showed very small ΔV values: DV = −4 and 2 cm^3^/mol for HepB2 and HepB3, respectively. The explanation could be that HepB1 and HepB3 can form only two-quartet GQs, while HepB2 is able to form a GQ consisting of three G-quartets. This theory correlates with the signs of the volume change values, which is negative only in the case of the three-quartet GQ.

Macgregor’s laboratory studied the telomeric GQ. They systematically investigated the effect of loops, cations, and nucleic acid concentration on the unfolding volume [154,155,156].

Although GQs can also be formed by RNAs, much less is known about RNA GQs, and their volumetric aspects have not yet been revealed.

Another of the important nucleic acid structures are the riboswitches. Sung and Nesbitt studied the conformational equilibrium of the lysine riboswitch using single-molecule FRET (smFRET) microscopy [67]. The conformational equilibrium of the lysine riboswitch shifts to the unfolded state as the pressure increases to 150 MPa. Increasing the lysine concentration stabilized the folded state, although the volume changes of the folding (Δ*V*_folding_ = 75 cm^3^/mol) did not change significantly. The unfolding pressure was in the range of 20–60 MPa, depending on the lysine concentration. This pressure range is the one relevant for deep-sea organisms. Therefore, it is noteworthy that TMAO (that is, an osmolyte commonly found in marine species, which has been shown to act as a chemical chaperone) can stabilize the folded state by reducing the Δ*V*_folding_ in a concentration-dependent manner.

The volumetric properties of protein–nucleic acid interactions can also be effectively investigated by smFRET [22]. After the successful characterization of the main molecular components of the living systems, the description of their interactions could be a promising direction.

### 5.4. Supramolecular Structures

#### 5.4.1. Viruses

Viruses have no cellular structure and contain one or more molecules of either RNA or DNA enclosed in a protein coat or capsid. Although the pressure behavior of a number of viruses has been investigated, the most interesting and dangerous ones can be treated only in special safe laboratories, and these safety measures hinder high pressure studies.

In the case of viruses, the aim of high pressure research is twofold [157]. First, the inactivation of viruses is an important aspect of food safety. In particular, shellfish, especially oysters, clams, and mussels, are susceptible to viral contamination [158]. On the other hand, inactivated viruses can gain possible application in vaccines [159].

The tobacco mosaic virus was the first virus subjected to pressure, but it was found quite pressure insensitive; 920 MPa was necessary for its inactivation [157]. As in the case of proteins, sub-denaturing the concentration of urea can help to solve this problem; the combination of urea and high pressure values of 100–300 MPa was successfully used to inactivate viruses including the tobacco mosaic virus [160].

Siva’s laboratory performed a series of experiments on viruses. They inactivated the foot-and-mouth disease virus (FMDV) using a treatment consisting of 2.5 kbar at −15 °C and 1 M urea, which completely abolished the infectivity of FMDV, while interestingly maintaining the integrity of its capsid structure [159].

The same group inactivated SA11-4S rotaviruses by more than five log units, subjecting them to high pressure. They proposed that the receptor-binding protein VP4 was altered by pressure in a similar way as the virus interacts with target cells [161].

Although the pressure-assisted inactivation of viruses is a proven method for possible vaccine applications, it is still an open question as to how it could compete with new-generation vaccine technologies.

#### 5.4.2. Bacteria

The inactivation of bacteria is a key problem in the food industry, where high pressure techniques can be employed. High-pressure treatment is considered as an alternative to thermal food processing technologies, as it has several advantages, e.g., better preservation of valuable food constituents, and it provides safe and high-quality food [162]. Although pressure inactivation by five log levels was achieved for several bacteria (*C. jejuni*, *E. coli*, *L. casei*, *L. monocytogenes* 74903, and *Z. balii*) using a five-minute treatment at 500 MPa [162], the pressurization (sometimes called pascalization, analogous to pasteurization) could not be as effective, as it is required for food safety regulations. This means it should be combined with other methods [163,164].

Another problem is the presence of bacterial spores, as these dried, dormant bacteria are very stable. A possible solution proposed for this issue consists of inducing the germination of these spores first, and then applying a second treatment to kill the sensitive germinated bacteria. That is how the pressure-induced germination process became the focus of numerous research studies [165]. During germination, dipicolinic acid (DPA) is released from the spores, which is a unique marker of germination. Germination can be detected by measuring the Raman spectroscopic signs of the Ca-DPA acid or by the high-pressure NMR of DPA [69,166]. Synchrotron infrared spectroscopy is another, more sophisticated, method used for the detection of the germination [167].

Despite the wide research activity in this field, practical applications, i.e., pressurized food products, are very rare, and they may only appear as special high-quality food products for a limited range of customers [162,168].

## 6. Summary

The application of high pressure spans a wide variety of systems and practical applications in the field of bioscience. Therefore, this review cannot be comprehensive in including all details, it focuses on the main and promising directions of the high pressure biological and biotechnological studies.

The high pressure behavior of several simple biological structures has already been characterized. However, the field cannot be treated as a closed scientific area. There are several promising directions, such as studying membranes containing unusual lipids or lipid mixtures which bring us closer to real biological membranes. The newly discovered exosomes also fit in this line. As for the proteins, the role of protein–protein interactions in supramolecular structures—like ribosomes or virus capsids—or the function of motor proteins under pressure are all largely uncovered scientific topics. In the field of nucleic acids, high pressure gained new possibilities upon the discovery of G-quadruplex structures, and there is still a lot to discover in this area. Life under pressure and adaptation to it in the deep sea is a largely unknown subject, where a lot of research has to be completed. The involvement of modern sequencing methods can also provide new insight into these questions.

From the point of technical development, the involvement of new experimental techniques, such as single-molecule microscopic methods, can open new directions for high pressure bioresearch.

In summary, I can state that high pressure bioscience remains an attractive research field in the future.

## Data Availability

Not applicable.
